# Warfarin sensitivity is associated with increased hospital mortality in critically Ill patients

**DOI:** 10.1371/journal.pone.0267966

**Published:** 2022-05-05

**Authors:** Zhiyuan Ma, Ping Wang, Milan Mahesh, Cyrus P. Elmi, Saeid Atashpanjeh, Bahar Khalighi, Gang Cheng, Mahesh Krishnamurthy, Koroush Khalighi

**Affiliations:** 1 Department of Medicine, St Luke’s University Health Network, Easton, PA, United States of America; 2 Department of Computer Science, East Carolina University College of Engineering and Technology, Greenville, NC, United States of America; 3 Drexel University College of Arts and Sciences, Philadelphia, PA, United States of America; 4 Lehigh University College of Arts and Sciences, Bethlehem, PA, United States of America; 5 Department of Biology, University of Hartford, West Hartford, CT, United States of America; 6 School of Pharmacy, Temple University, Philadelphia, PA, United States of America; 7 Division of Cardiology, Department of Medicine, University of Louisville School of Medicine, Louisville, KY, United States of America; 8 Lehigh Valley Heart Institute, Easton, PA, United States of America; Wroclaw University of Science and Technology, POLAND

## Abstract

**Background:**

Warfarin is a widely used anticoagulant with a narrow therapeutic index and large interpatient variability in the therapeutic dose. Warfarin sensitivity has been reported to be associated with increased incidence of international normalized ratio (INR) > 5. However, whether warfarin sensitivity is a risk factor for adverse outcomes in critically ill patients remains unknown. In the present study, we aimed to evaluate the utility of different machine learning algorithms for the prediction of warfarin sensitivity and to determine the impact of warfarin sensitivity on outcomes in critically ill patients.

**Methods:**

Nine different machine learning algorithms for the prediction of warfarin sensitivity were tested in the International Warfarin Pharmacogenetic Consortium cohort and Easton cohort. Furthermore, a total of 7,647 critically ill patients was analyzed for warfarin sensitivity on in-hospital mortality by multivariable regression. Covariates that potentially confound the association were further adjusted using propensity score matching or inverse probability of treatment weighting.

**Results:**

We found that logistic regression (AUC = 0.879, 95% CI: 0.834–0.924) was indistinguishable from support vector machine with a linear kernel, neural network, AdaBoost and light gradient boosting trees, and significantly outperformed all the other machine learning algorithms. Furthermore, we found that warfarin sensitivity predicted by the logistic regression model was significantly associated with worse in-hospital mortality in critically ill patients with an odds ratio (OR) of 1.33 (95% CI, 1.01–1.77).

**Conclusions:**

Our data suggest that the logistic regression model is the best model for the prediction of warfarin sensitivity clinically and that warfarin sensitivity is likely to be a risk factor for adverse outcomes in critically ill patients.

## Introduction

Warfarin is the most widely used oral anticoagulant worldwide. However, it has a narrow therapeutic window and large interpatient variability and incorrect warfarin dosing is associated with increased risk of bleeding or thromboembolism [1]. As a result, it is one of the leading common drugs implicated in emergency department visits and an important cause of drug-related mortality [2, 3]. Polymorphisms in cytochrome p450, family 2, subfamily C, polypeptide 9 (*CYP2C9*), and vitamin K epoxide reductase complex, subunit 1 (*VKORC1*) have been reported to be independently correlate with warfarin therapeutic dose [4–6]. Genetic variants in those two genes account for approximately 30% (20%-25% for *VKORC1* rs9923231; 5%-10% for *CYP2C9*) of the interpatient warfarin dose variability [4–7]. Many pharmacogenetic algorithms have been developed to predict the individual warfarin dose by integrating clinical, demographic, and genetic variables [8–11]. Due to the strong genetic effects on warfarin dose, the U.S. Food and Drug Administration (FDA) issued the warfarin product label to instruct how to initiate the individualized dose based on combined genetic variants of *CYP2C9* and *VKORC1* [12]. Despite these effects, a classification for warfarin responses in patients to reflect the genetic influence is needed. Recently, a previous study proposed a classification of warfarin sensitivity based on combined polymorphisms of *CYP2C9* and *VKORC1* and found that the average incidence of international normalized ratio (INR) >5 in the sensitive and very sensitive combined group was nearly 2-fold more frequent than that in the normal group, suggesting warfarin sensitive patients are more prone to bleeding complications [13]. However, whether warfarin sensitivity is a risk factor for adverse outcomes in critically ill patients remains unknown.

Machine learning that has been gradually appreciated and applied into clinical use, is to devise models and algorithms that lend themselves to prediction without being explicitly programmed. Data is fed to the machine learning algorithm, and the algorithm builds logic based on the data given. Machine learning models have been shown to easily identify trends and patterns, and handle multi-dimensional, multi-variety data and non-linear relationship [14]. In our previous study, we developed a clinical algorithm to predict the warfarin sensitivity based on logistic regression [13]. However, the performance of different machine learning algorithms for the prediction of warfarin sensitivity has yet to be determined for clinical applications.

In the present study, we aimed to evaluate the utility of different machine learning algorithms for the prediction of warfarin sensitivity and then to investigate the impact of warfarin sensitivity predicted using the best performance model on the outcomes of critically ill adult patients.

## Materials and methods

### Study population

A total of 106 qualified patients with various cardiovascular diseases on warfarin therapy in the Easton cohort (S1 File) has been reported [13]. This study protocol was approved by Copernicus Group Institutional Review Boards with a waiver of informed consent for retrospective analysis of de-identified data.

IWPC Cohort has been described previously [9, 15]. Expanded dataset was downloaded from the PharmGKB website (http://www.pharmgkb.org/downloads/), which contains pooled data on 6922 chronic warfarin users recruited through collaborative efforts of 22 research groups from 4 continents. This data set includes detailed de-identified curated data on demographic factors, clinical features, such as age, weight, height, and concomitant use of amiodarone, as well as *CYP2C9* and *VKORC1* genotypes. Missing values for height and weight were imputed with multivariate linear regression models. Specifically, weight, race, and sex were used for the imputation of the height variable, while height, race, and sex were used for the weight variable. For missing values of the *VKORC1* rs9923231, the imputation strategy has been described [9], which is based on linkage disequilibrium in *VKORC1* and race. We excluded those that did not have warfarin stable dose, missing age or lacking *CYP2C9* and *VKORC1* rs9923231 genotypes after imputation. We also excluded 17 subjects with CYP2C9*5, *6, *11, *13 and *14, due to low allele frequency and an outlier subject with warfarin stable dose 315 mg/week. A total of 5444 subjects were included in this study.

The dataset for investigating the impact of warfarin sensitivity on the outcomes of critically ill adult patients was extracted from the Medical Information Mart for Intensive Care IV (MIMIC-IV) version 0.4. MIMIC-IV is a large and freely available database containing de-identified health-related data associated with patients that stayed in critical care units at the Beth Israel Deaconess Medical Center between 2008 and 2019 [16]. The database was approved by the Institutional Review Boards of the Massachusetts Institute of Technology. One author (ZM) was given permission to extract data from MIMIC-IV. Patients who used warfarin during hospitalization were eligible for inclusion. For patients with multiple ICU admissions, we only included the first ICU stay. We excluded patients who were younger than 18 years old or ICU length of stay less than 1 day. The primary outcome was 28-day mortality from the date of ICU admission. Missing values for height were imputed as in the IWPC cohort.

### Warfarin sensitivity

Warfarin sensitivity has been defined in our previous study based on the FDA warfarin label (S1 Table) [12]. Briefly, *VKORC1* G/G; *CYP2C9* *1/*1, *VKORC1* G/G; *CYP2C9* *1/*2 and *VKORC1* A/G; *CYP2C9* *1/*1 were three compound genotypes for warfarin normal responders. The rest 15 compound genotypes were deemed warfarin sensitive including sensitive and very sensitive groups. In this study, warfarin sensitivity (normal or sensitive) was used as a categorical variable.

### The logistic model for predicting warfarin sensitivity

The logistical model to predict warfarin sensitivity followed our previously developed regression equation [13]: Probability (P) = 1–1 / [1 + exp(6.3606–1.0903$\sqrt{weekly\;warfarin\;stable\;dose}$ + 0.0075 × height + 0.0116 × weight– 0.2693 × age– 3.8913 × Black– 1.4203 × White– 1.9562 × Missing or mixed race– 0.6882 × amiodarone)]; where exp is the exponential function; height in cm; weight in kg; Age in decades; Black = 1 if race is Black, otherwise 0; White = 1 if race is White, otherwise 0; Missing or mixed race = 1 if race is unspecified or mixed, otherwise 0; amiodarone = 1 if patient taking amiodarone, otherwise 0. If P > 0.4, warfarin response is sensitive. It has been shown in our previous publication [13] that the accuracy, sensitivity, and specificity in the logistic regression is better with a threshold of probability > 0.4 for warfarin sensitivity. In the MIMIC-IV dataset, warfarin sensitivity was predicted by using the above logistic model. Warfarin stable dose was estimated from the most frequent daily dose prescribed during hospitalization.

### Statistical analysis

#### Implementation of machine learning algorithms and parameters

In the IWPC data set, we randomly chose 80% of the eligible patients (for a total of 4355) as the derivation cohort (training data set) to train various classifiers. The remaining 20% of the patients (N = 1089) were reserved as the validation cohort (testing data set) to calculate the estimates of correct classification rates. The variables were initially identified based on reported pharmacogenetic dosing algorithm [9], including warfarin stable dose, height, weight, race, age, and use of amiodarone. For the cross-validation (CV), the IWPC data set was randomly partitioned into 5 equal parts (folds). Thus, in each iteration, a model was trained on all but one held-aside folds and then tested on the held-aside fold of the data. The iteration was repeated 5 times and each fold served as a test data set to evaluate the model performance. For model quality assessment, the areas under the ROC Curve (AUC), the overall prediction accuracy and F1 score of the 5-fold CV were evaluated.

The neural networks (NN), random forest (RF), extremely randomized trees (ET), support vector machine with a linear kernel (SVC), AdaBoost (AB), K Nearest Neighbors (KNN), Logistic Regression (LG), Gaussian Naïve Bayes (GNB) were implemented using python library Scikit-learn (version 0.19.1) [17]. Gradient boosting trees (LGBT) was implemented using Microsoft’s software ‘LightGBM’ with a python wrapper [18]. Hyperparameter tuning was performed using ‘GridSearchCV’ with the default 5-fold CV in Scikit-learn. The key parameters for each algorithm were: hidden layer sizes = (200,) for NN; n_estimators = 250, 75, 50 and 25 for RF, ET, AB and LGBT, respectively; n_neighbors = 5, weights = ‘distance’ for KNN; kernel = ‘linear’, regularization C = 20, probability = ‘True’ for SVC. The performance of the logistic regression model was compared to other machine learning algorithms using unpaired Student’s t-test for 5-fold CV in the IWPC Cohort and McNemar’s χ2 test in the Easton cohort.

#### Multivariable regression

To investigate the potential impact of warfarin sensitivity on the primary outcome, multivariable regression was applied. Clinically relevant confounders including age, height, weight, gender, race, service units, simplified acute physiology score (SAPS) II, sequential organ failure assessment (SOFA) score, interventions (mechanical ventilation, vasopressor use, sedative use), comorbidities (congestive heart failure (CHF), coronary heart disease (CAD), asthma, chronic obstructive pulmonary disease (COPD), endocarditis, atrial fibrillation, chronic renal disease, chronic liver disease, respiratory failure, ARDS, pneumonia, stroke, and malignancy), clinical lab tests on first day of ICU stay (hemoglobin, platelet, WBC, bicarb, BUN, creatinine, chloride, sodium, potassium) and vital signs (mean blood pressure, respiratory rate, temperature, SpO2) were entered into a multivariate logistic regression model as covariates.

#### Propensity score matching (PSM) and inverse probability of treatment weighting (IPTW)

To account for the potential confounders associated with the predicted warfarin sensitivity and to ensure the robustness of our results, propensity score matching was used based on variables as described in the multivariable regression. Propensity scores for each patient were estimated by a multivariate logistic regression model. The matched cohort was created at a 1:1 with a caliper size of 0.05. Using the estimated propensity scores as weights, the IPTW method [19] was used to generate additional weighted cohort (IPTW cohort). The balance between covariates was evaluated by estimating standardized mean differences (SMD). SMD < 0.1 is considered a negligible group imbalance.

Due to the skewed distribution (with a longer tail at high doses) of warfarin dose, we transformed the raw dose into the square root of the dose. For differences in continuous variables, warfarin stable dose, height, and weight between the derivation and validation cohorts were compared with the Wilcoxon rank-sum test. For categorical variables, Fisher exact test was used in *CYP2C9* allele frequencies, χ^2^ tests were used for *VKORC1* rs9923231 genotype, age, and race. All the quantitative data are presented as means with 95% confidence intervals (CI) or medians with interquartile ranges. P values < 0.05 were considered to be statistically significant. All statistical analyses were conducted with R (version 3.6.1).

## Results

### Basic characteristics of study population

The characteristics of the patients in the IWPC and Easton cohorts are shown in S2 Table. In the Easton cohort, of 106 patients on long-term warfarin therapy for thromboembolic disorders and other cardiovascular diseases were included for analyses with complete clinical and genotype data. The median warfarin stable dose was 27.5 mg/week. In the IWPC cohort, 5444 patients were included for analyses with a median warfarin stable dose of 28.0 mg/week.

**Fig 1:**
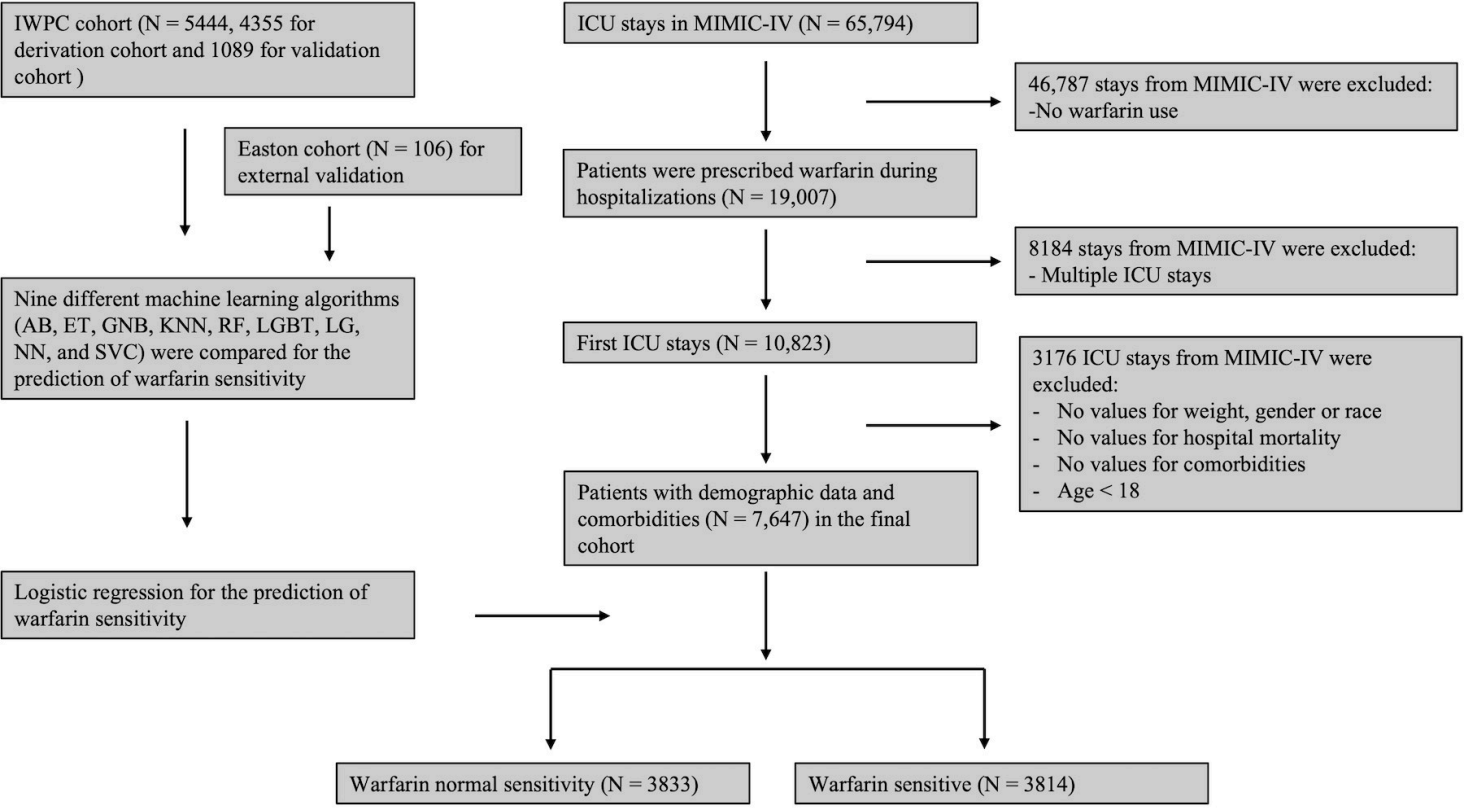
Flowchart illustration of the study cohorts. AB: AdaBoost, ET: Extremely Randomized Tree, GNB: Gaussian Naïve Bayes, KNN: K Nearest Neighbors, RF: Random Forests, LGBT: Light Gradient Boosting Tree, LG: Logistic Regression, NN: Neural Network, SVC: Support Vector Machine.

In the MIMIC-IV cohort, 19,007 patients were prescribed warfarin during hospitalizations and 10,823 patients were admitted to ICU with first stays. Based on exclusion criteria, a total of 7,647 critically ill patients were enrolled in the final cohort. There were 3833 patients predicted to be warfarin normal sensitivity and 3814 patients deemed as warfarin sensitive by our logistic model. The flow diagram of patient selections was shown in Fig 1. The baseline characteristics of MIMIC-IV cohort were summarized in Table 1.

**Table 1:** Comparisons of demographic and clinical characteristics between the original cohort and matched cohort.

Covariates	Original cohort			Matched cohort		
	Normal	Sensitive	SMD	Normal	Sensitive	SMD
N	3833	3814		2851	2851	
Age (Median)	71	72	0.013	71	73	0.003
Male (%)	59.2	58.6	0.013	60.3	62.6	0.048
Weight-kg			0.013			0.016
Median	82.5	81.5		82.5	81.4	
Interquartile range	70.0–98.0	68.1–97.6		69.9–97.6	68.9–97.0	
Height-m			0.041			0.026
Median	172.7	170.2		172.7	172.7	
Interquartile range	162.0–177.7	161.1–177.8		161.6–177.8	162.6–177.8	
Race-no. (%)			0.644			0.010
Asian	6 (0.2)	128 (3.4)		6 (0.2)	6 (0.2)	
Black	585 (15.3)	33 (0.9)		33 (1.2)	33 (1.2)	
White	2500 (65.2)	3137 (82.2)		2324 (81.5)	2313 (81.1)	
Unknown	742 (19.4)	516 (13.5)		488 (17.1)	499 (17.5)	
Service unit-no. (%)			0.232			0.149
CCU	913 (23.8)	739 (19.4)		638 (22.4)	646 (22.7)	
CSICU	784 (20.5)	1013 (26.6)		692 (24.3)	843 (29.6)	
MICU	1194 (31.2)	1266 (33.2)		868 (30.4)	750 (26.3)	
SICU	233 (6.1)	263 (6.9)		183 (6.4)	171 (6.0)	
Others	709 (18.5)	533 (14.0)		470 (16.5)	441 (15.5)	
SAPSII	35.7 (12.3)	37.1	0.113	36.4	36.4	0.001
SOFA	4.7 (3.2)	5.3 (3.4)	0.194	4.87	4.97	0.033
Interventions- (%)						
Ventilation	36	41	0.091	39	38	0.023
Vasopressor use	42	49	0.144	46	49	0.069
Sedative use	71	75	0.101	73	75	0.049
Comorbidity- (%)						
Endocarditis	1	2	0.032	1	2	0.029
CHF	39	41	0.039	39	38	0.019
CAD	36	38	0.049	38	39	0.030
COPD	14	15	0.049	14	14	0.010
AFIB	56	61	0.102	60	62	0.039
Renal	24	25	0.038	22	22	0.022
Liver	3	4	0.076	3	3	0.006
Resp fail	18	20	0.051	18	17	0.042
ARDS	3	3	0.041	3	3	0.019
Pneumonia	16	17	0.025	16	15	0.019
Stroke	8	6	0.067	7	7	0.001
Malignancy	12	11	0.016	12	10	0.037
Vital signs-Mean (SD)						
HR (/min)	84.1 (15.9)	84.3 (15.4)	0.013	83.9 (15.6)	83.5 (15.2)	0.027
RR (/min)	19.2 (3.5)	19.1 (3.5)	0.022	19.1 (3.5)	19.0 (3.5)	0.019
MBP (mmHg)	77.9 (10.5)	76.0 (10.0)	0.183	76.8 (9.8)	76.7 (10.0)	0.008
Temperature (°C)	36.8 (0.5)	36.8 (0.5)	0.016	36.8 (0.5)	36.8 (0.5)	0.025
SpO2	96.9 (1.8)	96.9 (1.9)	0.006	96.9 (1.8)	96.9 (1.9)	0.029
Laboratory tests-Mean (SD)						
Hemoglobin (g/L)	11.6(2.1)	11.4 (2.0)	0.094	11.6 (2.0)	11.6 (2.0)	0.002
Platelet (x10^9^/L)	228.5 (110.6)	224.5 (111.0)	0.036	224.9 (105.7)	221.2 (104.0)	0.035
WBC (x10^9^/L)	13.6 (7.0)	14.4 (8.9)	0.109	14.0 (7.3)	14.3 (9.3)	0.041
Bicarbonate (mmol/L)	25.1 (4.0)	24.9 (4.1)	0.067	25.1 (4.1)	25.0 (3.9)	0.012
Chloride (mmol/L)	105.6 (6.1)	105.6 (6.0)	0.008	105.7 (6.0)	105.9 (5.8)	0.032
Sodium (mmol/L)	139.6 (4.3)	139.3 (4.4)	0.063	139.4 (4.2)	139.4 (4.2)	0.005
Potassium (mmol/L)	4.6 (0.8)	4.6 (0.8)	0.030	4.6 (0.8)	4.6 (0.7)	0.013
BUN (mg/dL)	29.3 (22.6)	30.7 (24.0)	0.058	29.3 (22.2)	28.7 (22.3)	0.027
Creatinine (mg/dL)	1.6 (1.7)	1.6 (1.5)	0.014	1.5 (1.4)	1.5 (1.3)	0.030
ALT (Tested %)	45.0	45.0	0.001	43.5	41.2	0.046
TB (Tested %)	44.6	45.6	0.020	43.8	41.3	0.050
CK (Tested %)	37.2	32.5	0.098	34.2	33.2	0.021

AFIB, atrial fibrillation; ALT, alanine aminotransferase; ARDS, acute respiratory distress syndrome; BUN, blood urea nitrogen; CHF, congestive heart failure; CAD, coronary artery disease; CK, creatine kinase; COPD, chronic obstructive pulmonary disease; HR, heart rate; Liver, chronic liver disease; MBP, mean blood pressure; Renal, chronic renal disease; Resp fail, respiratory failure; RR, respiratory rate; SAPSII, simplified acute physiology score II; SOFA, sequential organ failure assessment score; SMD, standardized mean difference; SpO2, pulse oxygen saturation; TB, total bilirubin; WBC, white blood cell.

### Performance of the different algorithms

**Fig 2:**
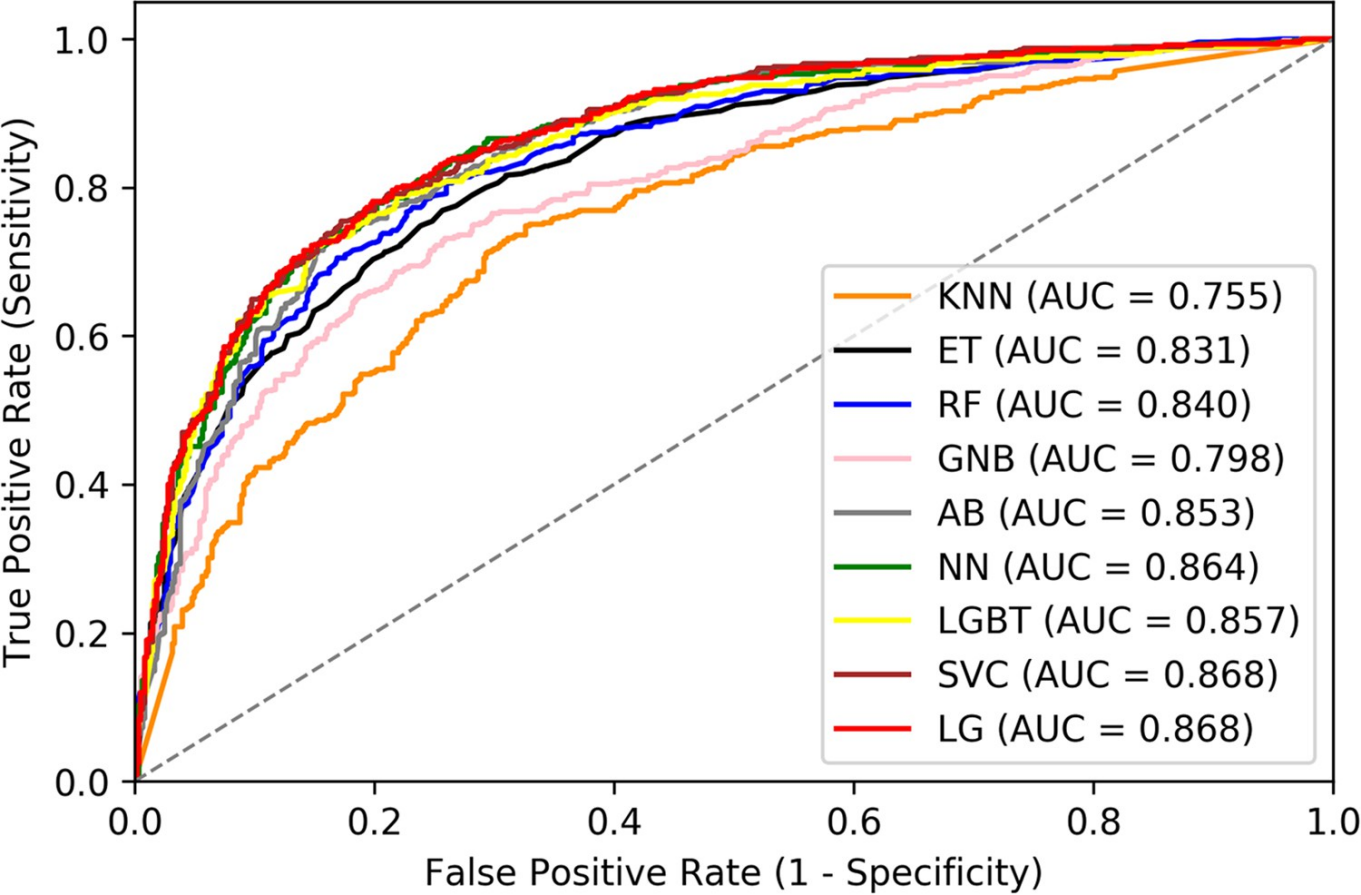
ROC curve analysis of nine different machine learning algorithms using the validation cohort in the IWPC cohort. AB: AdaBoost, ET: Extremely Randomized Tree, GNB: Gaussian Naïve Bayes, KNN: K Nearest Neighbors, RF: Random Forests, LGBT: Light Gradient Boosting Tree, LG: Logistic Regression, NN: Neural Network, SVC: Support Vector Machine.

Previously, we developed a clinical algorithm to predict warfarin sensitivity based on logistic regression [13]. To determine whether the performance of different machine learning algorithms is better than logistic regression, the same features were selected to train the various prediction classifiers using the derivation data set from the IWPC cohort. There was no statistically significant difference between the derivation and validation data set (S2 Table). The maximal predictive performance evaluated with the validation data set was obtained with the logistic regression (AUC = 0.868) and SVC (AUC = 0.868), followed by NN (AUC = 0.864) and LGBT (AUC = 0.857), and AB (AUC = 0.853) (Fig 2). The algorithms RF, ET, GNB and KNN resulted in the least favorable results (Fig 1). To obtain robust results, the model performance was evaluated by 5-fold CV (Table 2). Similarly, the algorithm logistic regression (AUC = 0.879, 95% CI: 0.834–0.924; Accuracy = 0.754, 95% CI: 0.649–0.859; F1 score = 0.691, 95% CI: 0.415–0.963) was indistinguishable from SVC, NN, LGBT and AB (P > 0.05).

**Table 2:** AUC, accuracy and F1 score for different machine learning algorithms using 5-fold CV in the IWPC cohort.

Algorithms	AUC		Accuracy		F1 score	
	Mean(95% CI)	P value (vs. LG)	Mean (95% CI)	P value (vs. LG)	Mean (95% CI)	P value (vs. LG)
**RF**	0.830(0.795–0.865)	0.010	0.733(0.671–0.795)	0.523	0.681(0.491–0.871)	0.904
**ET**	0.826 (0.785–0.866)	0.009	0.729 (0.677–0.781)	0.441	0.676 (0.494–0.859)	0.864
**AB**	0.863 (0.834–0.892)	0.289	0.751 (0.674–0.828)	0.944	0.702 (0.504–0.901)	0.902
**NN**	0.874 (0.826–0.922)	0.782	0.745 (0.608–0.882)	0.856	0.645 (0.253–1.037)	0.714
**LGBT**	0.857 (0.830–0.884)	0.134	0.746 (0.662–0.830)	0.828	0.689 (0.456–0.921)	0.978
**KNN**	0.729 (0.648–0.810)	<0.001	0.668 (0.606–0.730)	0.025	0.617 (0.442–0.792)	0.891
**GNB**	0.828 (0.725–0.932)	0.117	0.671 (0.551–0.792)	0.079	0.686 (0.634–0.737)	0.937
**SVC**	0.877 (0.835–0.919)	0.916	0.750 (0.647–0.852)	0.920	0.684 (0.399–0.969)	0.945
**LG**	0.879 (0.834–0.924)		0.754 (0.649–0.859)		0.691 (0.415–0.968)	

P values for the difference between logistic regression and other algorithms were calculated by the Student’s t test.

AB: AdaBoost, ET: Extremely Randomized Tree, GNB: Gaussian Naïve Bayes, KNN: K Nearest Neighbors, RF: Random Forests, LGBT: Light Gradient Boosting Tree, LG: Logistic Regression, NN: Neural Network, SVC: Support Vector Machine.

### Feature importance

**Fig 3:**
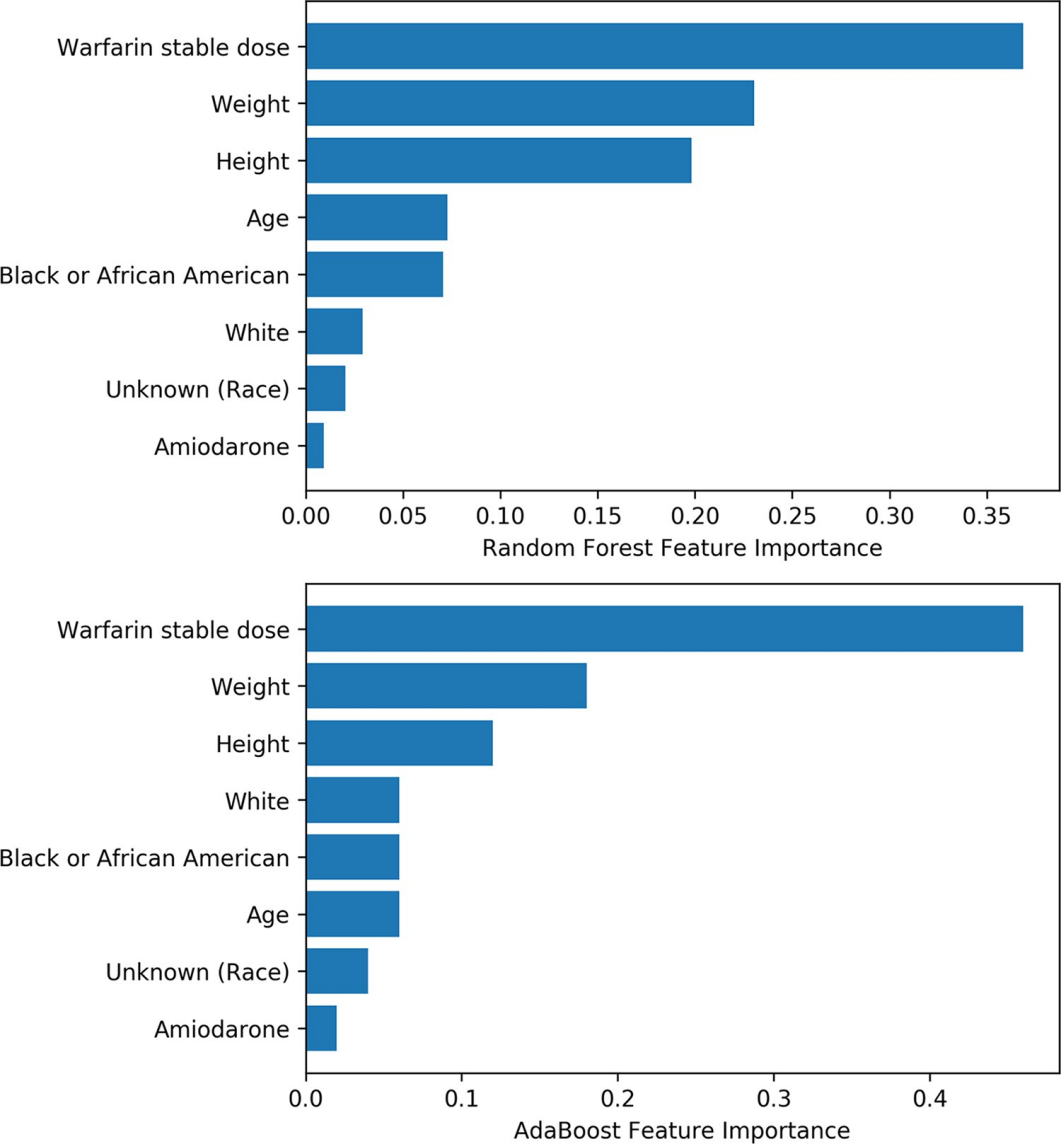
The importance of features in the random forest and AdaBoost machine learning models.

To investigate the importance of selected features in the prediction models, the relative feature importance was ranked by random forest and AdaBoost algorithms using the IWPC cohort. The warfarin stable dose was the most important variable to predict warfarin sensitivity (Fig 3).

### Validation of the performance

To fully utilize the IWPC data set, we pooled the derivation and validation cohorts to train the nine different classifiers, and then validated the performance of the various machine learning algorithms using the external Easton cohort. As shown in Table 3, consistent with the results in the IWPC cohort, the classifier logistic regression (AUC = 0.835; Accuracy = 0.802; and F1 score = 0.759) was non-inferior to SVC, NN, AB and LGBT (P > 0.05), and better than all the other algorithms (P < 0.05).

**Table 3:** AUC, accuracy and F1 score for different machine learning algorithms tested in the Easton cohort.

Algorithms	AUC	Accuracy	F1 score	P value (vs. LG)
**RF**	0.748	0.698	0.644	0.006
**ET**	0.748	0.670	0.628	0.006
**AB**	0.826	0.755	0.717	0.131
**NN**	0.824	0.783	0.747	0.617
**LGBT**	0.815	0.755	0.723	0.131
**KNN**	0.715	0.689	0.629	0.034
**GNB**	0.736	0.623	0.672	0.002
**SVC**	0.836	0.802	0.759	NA*
**LG**	0.835	0.802	0.759	

P values for the difference between logistic regression and other algorithms were calculated using the McNemar’s χ2 test.

* P value could not be calculated due to the exact same prediction.

AB: AdaBoost, ET: Extremely Randomized Tree, GNB: Gaussian Naïve Bayes, KNN: K Nearest Neighbors, RF: Random Forests, LGBT: Light Gradient Boosting Tree, LG: Logistic Regression, NN: Neural Network, SVC: Support Vector Machine.

### Warfarin sensitivity and hospital mortality

**Fig 4:**
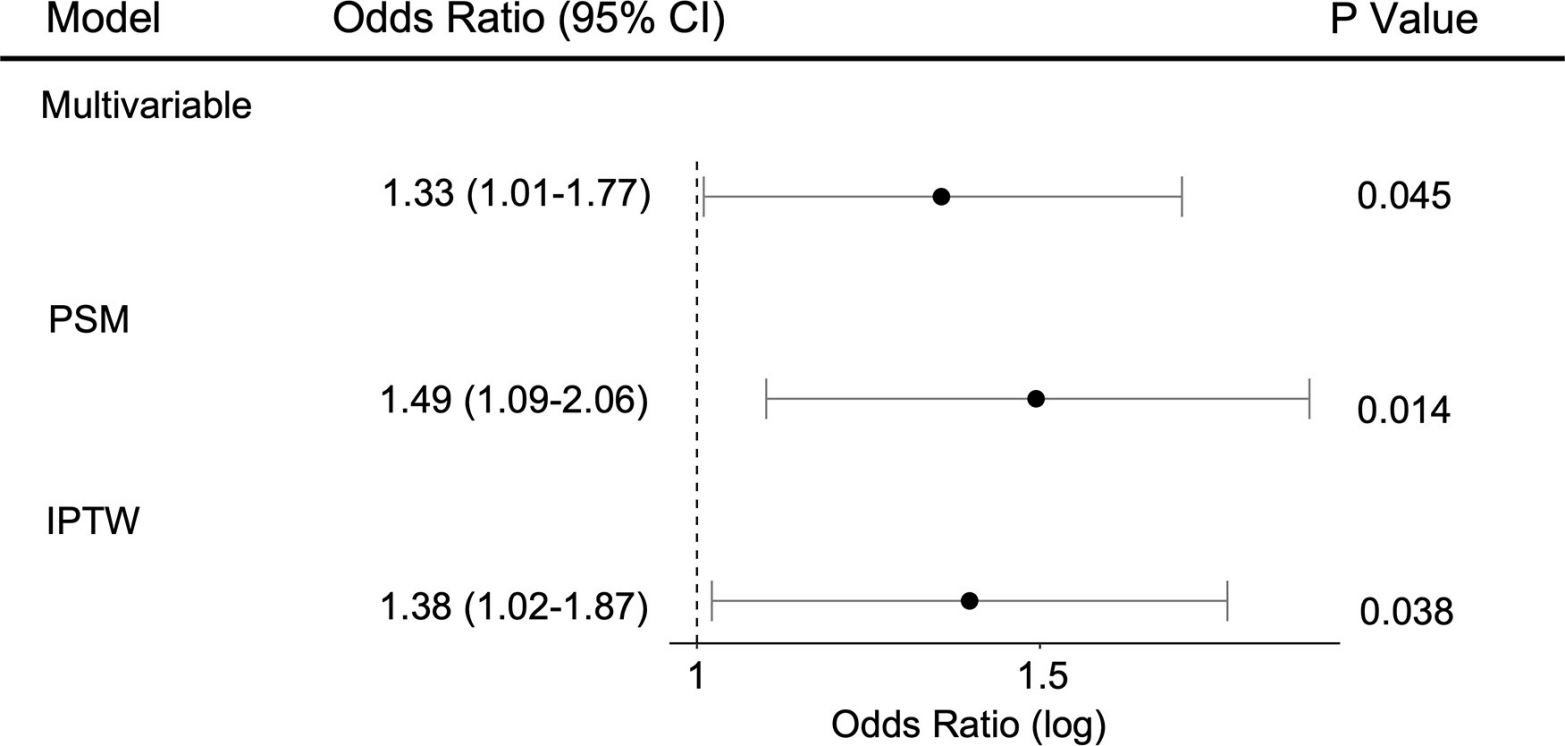
Primary outcome analysis with three different models. Warfarin sensitivity was significantly associated with increased in-hospital mortality. PSM, propensity score matching; IPTW, inverse probability of treatment weighting.

To determine the impact of warfarin sensitivity on critically ill patients during hospitalization, 28-day mortality since ICU admission was designated as the primary outcome. Of the 3814 in the warfarin sensitive group, primary outcome events occurred in 158 patients (4.14%), compared with 117 of 3833 (3.05%) in the warfarin normal group. In the multivariate logistic regression analyses, after adjusting age, height, weight, gender, race, service unit, SAPS score, SOFA score, interventions, comorbidities, clinical lab tests and vital signs on admission to ICU, warfarin sensitivity was significantly associated with higher primary outcome events (OR, 1.33; 95% CI, 1.01–1.77; P = 0.045) compared to normal warfarin sensitivity. To account for confounding that could lead to the protective association between warfarin sensitivity and the primary outcome, we further performed PSM and propensity score-based inverse probability of treatment weighting (IPTW) analyses. In the PSM matched cohort, after adjusting the above variables, there was an increased risk of primary outcome events in the warfarin sensitive group (adjusted OR, 0.1.49; 95% CI, 1.09–2.06; P = 0.014) (Fig 4). Similarly, in the IPTW matched cohort, the adjusted OR for the primary outcome was 1.38 (95% CI, 1.02–1.87; P = 0.038).

## Discussion

Direct oral anticoagulants (DOACs), also known as non-vitamin K antagonist oral anticoagulants (NOACs), which have a wide therapeutic window, thereby facilitating fixed dosing in adults without the need for laboratory monitoring or dose adjustments for body weight, are now available as a possible alternative to warfarin. However, warfarin has proven efficacy, low cost, and years of physician experience compared with DOACs [20]. Warfarin offers superior efficacy compared to DOACs in high-risk patients with antiphospholipid syndrome and mechanical valves. The clinical trial for the use of rivaroxaban versus warfarin in patients with antiphospholipid syndrome was terminated prematurely and showed that an increased rate of events with rivaroxaban compared with warfarin [21]. The RE-ALIGN trial for the use of dabigatran versus warfarin in patients with mechanical heart valves was also terminated prematurely due to an excess of both thromboembolic events and bleeding events among patients in the dabigatran group [22]. In addition, warfarin may be a superior option for patients with a history of medication nonadherence or morbid obesity. Therefore, warfarin will remain an important and frequently used anticoagulant.

The individual response to warfarin is highly variable, being greatly influenced by genetic variants, for example, in *CYP2C9* and *VKORC1*. In view of the importance of genetic influence, we have proposed a classification for the individual response to warfarin and created a simple algorithm to predict warfarin sensitivity based on logistic regression [13]. Logistic regression is a widely used traditional statistical approach, but it is liable to ‘sparse-data biases’ in case of low cardinality of records especially when training and testing procedures are applied. Recently, machine learning algorithms are gradually appreciated and applied into clinical use in general medicine [23, 24]. In data analytics, machine learning is to devise models and algorithms that lend themselves to prediction. Machine learning can handle complex, nonlinear relationships among variables and deal with many sources of inferential trouble such as outliers and collinearity compared to linear methods, for instance, multivariable linear regression. It is therefore very necessary to compare various machine learning algorithms with logistic regression for the prediction of warfarin sensitivity.

In this study, using distinct machine learning algorithms, we developed eight models with the same variables as in the logistic regression model to predict warfarin sensitivity. Intriguingly, we found that the models produced by logistic regression was indistinguishable from SVC with a linear kernel, NN, AdaBoost and LGBT and significantly outperformed all the other algorithms in both the IWPC cohort and the external Easton cohort. This is consistent with the IWPC study, in which linear regression outperforms other machine learning-based algorithms for the prediction of warfarin maintenance dose [9]. The result has also been confirmed in Chinese patients that the performance of the linear regression model is superior [25]. This is probably attributed to the fact that machine learning algorithms excel at complex and non-linear models with many independent variables. Because the logistic regression model uses fewer variables, is easy to be implemented in a clinical setting and has the superior performance, it is the best model for the prediction of warfarin sensitivity clinically. However, the models in our study were relatively simple, only six variables included in our models for the prediction of warfarin sensitivity. Additional potential variables affecting the prediction of warfarin sensitivity were not included in the model, such as comorbidities, additional drug–drug interactions, and patient behaviors, including diet, exercise, and compliance. With more variables integrated into models, machine learning based models are likely to have better performance at the risk of overfitting.

It has been shown that predicted warfarin maintenance dose decreases as with the increase of age [26]. In the present study, we revealed that age was an important factor in the prediction of warfarin sensitivity. These data indicate age is linked to warfarin maintenance dose and sensitivity. In addition, we have shown that Asians, Whites, and Blacks have different polymorphism profiles of *CYP2C9* and *VKORC1* in the IWPC cohort [13]. In line with this, we found that race was another important variable to predict warfarin sensitivity.

Lastly, we applied the logistic regression model to predict warfarin sensitivity on critically ill patients in the MIMIC-IV database. Intriguingly, warfarin sensitive patients with critical illnesses were significantly associated with worse in-hospital mortality, compared to warfarin normal patients. This result suggests that warfarin sensitivity is a risk factor in critically ill patients.

Several limitations are present in our study. First, missing genotypes of *VKORC1* were derived based on linkage disequilibrium in the IWPC cohort. Missing values for height and weight were also imputed using multivariate linear regression. Errors could have been introduced in the study despite these generally reliable imputation strategies. Second, although potential confounding factors were attempted to balance and control by multiple variable adjustments and propensity score matching, due to the inherent nature of retrospective studies, residual confounders were likely to exist and could not be balanced in the analysis of the association of warfarin sensitivity and in-hospital mortality.

## Conclusions

We evaluated the utility of different machine learning algorithms for the prediction of warfarin sensitivity and found that logistic regression was indistinguishable from other machine algorithms such as SVC with a linear kernel, NN, AdaBoost and LGBT. We found that the logistic regression model is the best model for the prediction of warfarin sensitivity clinically. We also demonstrated that warfarin sensitivity predicted by the logistic regression model was significantly associated with increased in-hospital mortality in critically ill patients, suggesting warfarin sensitivity may be a risk factor for adverse outcomes in critically ill patients.

## Supporting information

S1 Table Warfarin sensitivity based on genotypes and three ranges of recommended warfarin doses (mg/day) from the USA FDA drug label (COUMADIN, Reference ID: 3022954). (DOCX)

S2 Table Demographic and clinical characteristics of the derivation, validation and Easton cohorts. (DOCX)

S1 File Easton cohort dataset. (XLSX)
