# Supplementary material for: Warfarin sensitivity is associated with increased hospital mortality in critically Ill patients
Source: PLoS One. 2022 May 5;17(5):e0267966. doi: 10.1371/journal.pone.0267966 (PMC9070894; doi:10.1371/journal.pone.0267966)
Supplement: S1 Table — (DOCX) [file pone.0267966.s001.docx]

**S1 Table. Warfarin sensitivity based on genotypes and three ranges of recommended warfarin doses (mg/day) from the USA FDA drug label (COUMADIN, Reference ID: 3022954).**

| **VKORC1** | **CYP2C9** | | | | | |
| --- | --- | --- | --- | --- | --- | --- |
|  | ***1/*1** | ***1/*2** | ***1/*3** | ***2/*2** | ***2/*3** | ***3/*3** |
| **G/G** | Normal  (5-7) | Normal  (5-7) | Sensitive  (3-4) | Sensitive  (3-4) | Sensitive  (3-4) | Very Sensitive  (0.5-2) |
| **A/G** | Normal  (5-7) | Sensitive  (3-4) | Sensitive  (3-4) | Sensitive  (3-4) | Very Sensitive  (0.5-2) | Very Sensitive  (0.5-2) |
| **A/A** | Sensitive  (3-4) | Sensitive  (3-4) | Very Sensitive  (0.5-2) | Very Sensitive  (0.5-2) | Very Sensitive  (0.5-2) | Very Sensitive  (0.5-2) |

Ranges are derived from multiple published clinical studies. VKORC1-1639G>A (rs9923231) variant is used in this table. Other co-inherited VKORC1 variants may also be important determinants of warfarin dose.
