# Supplementary material for: Warfarin sensitivity is associated with increased hospital mortality in critically Ill patients
Source: PLoS One. 2022 May 5;17(5):e0267966. doi: 10.1371/journal.pone.0267966 (PMC9070894; doi:10.1371/journal.pone.0267966)
Supplement: S2 Table — (DOCX) [file pone.0267966.s002.docx]

**S2 Table. Demographic and clinical characteristics of the Derivation, Validation and Easton cohorts.**

| **Variable** | **IWPC Cohort** | | |  |
| --- | --- | --- | --- | --- |
|  | **Derivation Cohort (N=4355)** | **Validation Cohort (N=1089)** | **P Value^*^** | **Easton Cohort**  **(N=106)** |
| Warfarin dose—mg/week |  |  | 0.17 |  |
| Median | 29.0 | 28.0 |  | 27.5 |
| Interquartile range | 20.5-40.3 | 21.0-40.0 |  | 17.5-37.9 |
| Genotype—no. (%) |  |  |  |  |
| *VKORC1* rs9923231 |  |  | 0.68 |  |
| G/G | 1464 (33.6) | 357 (32.8) |  | 35 (33.0) |
| A/G | 1593 (36.6) | 414 (38.0) |  | 54 (50.9) |
| A/A | 1298 (29.8) | 318 (29.2) |  | 17 (16.0) |
| *CYP2C9* |  |  | 0.18 |  |
| *1/*1 | 3281 (75.3) | 822 (75.5) |  | 62 (58.5) |
| *1/*2 | 583 (13.4) | 148 (13.6) |  | 21 (19.8) |
| *1/*3 | 381 (8.7) | 90 (8.3) |  | 19 (17.9) |
| *2/*2 | 51 (1.2) | 6 (0.6) |  | 1 (0.1) |
| *2/*3 | 47 (1.1) | 20 (1.8) |  | 3 (2.8) |
| *3/*3 | 12 (0.3) | 3 (0.3) |  | 0 (0) |
| Age—no. (%) |  |  | 0.93 |  |
| < 50 | 727 (16.7) | 187 (17.2) |  | 2 (1.9) |
| 50-80 | 3093 (71.0) | 770 (70.7) |  | 56 (52.8) |
| > 80 | 535 (12.3) | 132 (12.1) |  | 48 (45.3) |
| Height—m |  |  | 0.36 |  |
| Median | 167.6 | 167.6 |  | 170.2 |
| Interquartile range | 160.0-175.8 | 160.0-175.8 |  | 162.6-175.3 |
| Weight—kg |  |  |  |  |
| Median | 76.0 | 75.9 | 0.66 | 80.5 |
| Interquartile range | 63.0-90.9 | 63.5-90.0 |  | 69.9-94.3 |
| Race—no. (%) |  |  | 0.36 |  |
| White | 2334 (53.6) | 616 (56.6) |  | 106 (100) |
| Asian | 1148 (26.4) | 269 (24.7) |  |  |
| Black | 512 (11.8) | 117 (10.7) |  |  |
| Mixed or missing | 316 (7.3) | 87 (8.0) |  |  |
| Amiodarone | 204 (4.7) | 51 (4.7) | 1.0 | 10 (9.4) |
| Warfarin sensitivity—no. (%) | 1949 (44.8) | 485 (44.5) | 0.90 | 47 (44.3) |

* P values for the difference between the derivation and validation cohorts were calculated with the use of the Wilcoxon rank-sum test for warfarin dose, height, and weight, Fisher’s exact test for *CYP2C9* genotype, χ^2^ tests were used for *VKORC1* rs9923231 genotype, age, and race, and the z test for proportions for the use of amiodarone and warfarin sensitivity.
